# Transmission of a *Gammabaculovirus* within Cohorts of Balsam Fir Sawfly (*Neodiprion abietis*) Larvae

**DOI:** 10.3390/insects3040989

**Published:** 2012-10-19

**Authors:** Roger Graves, Dan T. Quiring, Christopher J. Lucarotti

**Affiliations:** 1 Faculty of Forestry and Environmental Management, P.O. Box 4400, The University of New Brunswick, Fredericton, New Brunswick, E3B 5A3, Canada; E-Mails: rogergraves19@gmail.com (R.G.); quiring@unb.ca (D.T.Q.); 2 Natural Resources Canada, Canadian Forest Service - Atlantic Forestry Centre, 1350 Regent Street, Fredericton, New Brunswick, E3C 2G6, Canada

**Keywords:** *Gammabaculovirus*, *Neodiprion abietis*, nucleopolyhedrovirus, balsam fir sawfly, *Abies balsamea*, disease transmission, pest management

## Abstract

Nucleopolyhedroviruses (NPV: *Gammabaculovirus: Baculoviridae*) of diprionid sawflies (Diprionidae: Hymenoptera) are highly host specific and only infect the midgut epithelium. While still alive, infected sawfly larvae excrete NPV-laden diarrhea that contaminates food sources. The diarrhea can then be consumed by conspecific larvae, resulting in rapid horizontal transmission of the virus. To better understand the efficacy of *Gammabaculovirus*-based biological control products, the horizontal spread of such a virus (NeabNPV) within cohorts of balsam fir sawfly (*Neodiprion abietis*) larvae was studied by introducing NeabNPV-treated larvae into single-cohort groups at densities similar to those observed during the increasing (field study) and peak (laboratory study) phases of an outbreak. In field studies (~200 *N. abietis* larvae/m^2^ of balsam fir (*Abies balsamea*) foliage), NeabNPV-induced mortality increased positively in a density-dependent manner, from 23% (in control groups) to 51% with the addition of one first-instar NeabNPV-treated larva, to 84% with 10 first–instar-treated larvae. Mortality was 60% and 63% when one or 10 NeabNPV-treated third-instar larva(e), respectively, were introduced into groups. Slightly higher levels of NeabNPV-induced mortality occurring when NeabNPV-treated larvae were introduced into first- rather than third-instar cohorts suggests that early instars are more susceptible to the virus. In the laboratory (~1330 *N. abietis* larvae/ m^2^ of foliage), NeabNPV-caused mortality increased from 20% in control groups to over 80% with the introduction of one, five or 10 NeabNPV-treated larvae into treatment groups of first-instar larvae.

## 1. Introduction

In North America, many diprionid sawflies (Diprionidae: Hymenoptera) are defoliators of coniferous trees and represent some of the most economically important pests of forest trees [1,2]. Typically, diprionid population outbreaks are of short duration, lasting only 3–5 yr, and are brought back to low density levels by natural enemies, especially nucleopolyhedroviruses (NPV) (*Gammabaculovirus*, *Baculoviridae*; [3]) [1,2,4].

Since the early 1990s, western Newfoundland (Newfoundland and Labrador (NL), Canada) has experienced the largest outbreak of balsam fir sawfly (*Neodiprion abietis* Harris) populations on record. Tens of thousands of hectares (ha) of forests dominated by balsam fir (*Abies balsamea* (L.) Mill.) have been impacted annually [5,6]. Historically, natural declines in balsam fir sawfly populations in western Newfoundland have been associated with epizootics of a species-specific NPV, NeabNPV [5,7,8,9]. NeabNPV has been demonstrated to be an effective control agent [10,11,12] as NeabNPV infection most often results in the death of infected balsam fir sawfly larvae [13].

Sawfly NPV infections are initiated when viral occlusion bodies (OBs) are ingested by feeding larvae. The proteinaceous OBs dissolve in the larval midgut, releasing the virions that attach to and enter midgut epithelial cells. In sawflies, NPV infections are limited to the midgut [14,15]. Horizontal transmission (between individuals of the same generation) of sawfly NPVs occurs through the continuous release of OBs from infected epithelial cells, which slough off the midgut and pass directly into the larval frass and, subsequently, contaminate the foliage where other conspecific larvae can consume them [15,16].

To better understand the efficacy of *Gammabaculovirus*-based biological control products, the horizontal spread of such a virus (NeabNPV) within cohorts of *N. abietis* larvae was studied by introducing NeabNPV-treated larvae into single-cohort groups at densities similar to those observed during the increasing (field study) and peak (laboratory study) phases of an outbreak. Additionally, because later stadial stages tend to confer greater resistance to pathogens [17,18], we investigated whether larval age when NeabNPV-treated larvae were introduced into a cohort of larvae influenced NeabNPV transmission in the field. 

## 2. Results and Discussion

### 2.1. Field Study

**Figure 1:**
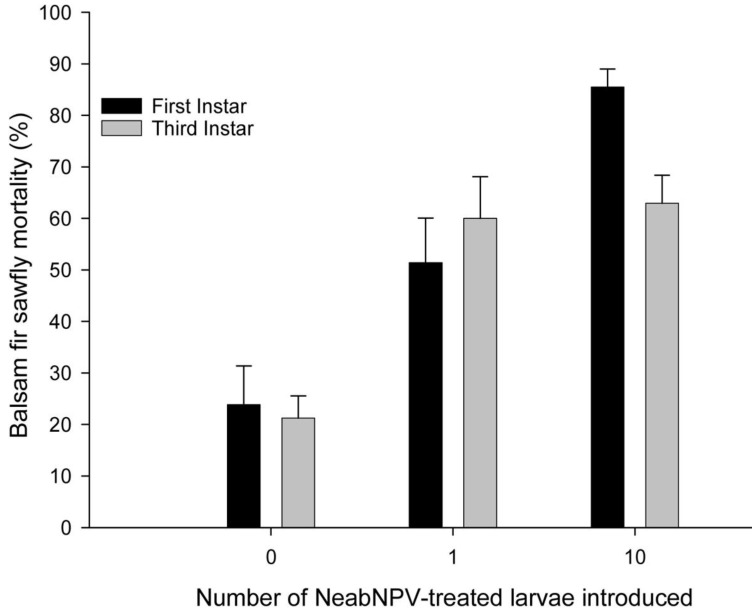
Mean (±SEM) larval mortality resulting from introducing one or 10 NeabNPV-infected *N. abietis* larvae into groups of 50 first or third instars feeding on branches of balsam fir foliage individually enclosed in sleeve cages in the field. Each treatment (10 replicate groups each [*i.e.*, first or third instars, zero (control), one or 10 larvae treated with NeabNPV and returned to bring each population back up to 50 larvae (3000 larvae on 60 branches)]) contained a total of 50 larvae at an equivalent density of approximately 220 larvae/m^2^ balsam fir foliage.

In the field experiments, mortality due to NeabNPV was ≤23% in control groups, but mortality exceeded 50% in cohorts into which NeabNPV-treated larvae were introduced (Figure 1). Larval mortality was significantly influenced by the number (Wald chi-square = 683.0, *p* < 0.001) and the instar (Wald chi-square = 46.6, *p* < 0.001) of NeabNPV-treated larvae introduced into cohorts. The mortality of larvae exposed to NeabNPV as third instars was 62.0 ± 4.5% (mean ± SEM), lower than 72.2 ± 4.8% for larvae exposed as first instars. The number of infected larvae introduced into a cohort influenced mortality more for first- than third-instar cohorts (Figure 1), resulting in a significant interaction between larval instar at the time of NeabNPV exposure and number of NeabNPV-treated larvae introduced into a cohort (Wald chi-square = 56.4, *p* < 0.001). 

Larval densities in the field experiment approximated mean branch densities in epidemic balsam fir sawfly populations [10,19]. However, the spatial distribution of early instars is very clumped in nature [20] and individual branches with densities similar to that used in the field study are most common during the increasing phase of balsam fir sawfly outbreaks. Generally, mean larval mortality attributable to NeabNPV infection increased with the number of infected larvae introduced into a cohort, but mortality decreased slightly when infected larvae were introduced into cohorts of third-*versus* first-instar larvae. First-instar cohorts may have had slightly higher rates of NeabNPV-induced mortality than third-instar cohorts due to increased time for multiple cycles of NeabNPV replication and subsequent release of OBs to occur, and/or because of higher susceptibility of first instars [17,18,21]. Increased levels of infection and subsequent mortality of larvae in cohorts into which 10 NeabNPV-treated larvae were introduced were presumably due to much higher levels of NeabNPV OB inoculum than in cohorts where only one treated larva was introduced. Although not statistically significant, the influence of the number of infected larvae introduced into a cohort on larval mortality appeared to be more important for first than third instars (Figure 1), perhaps due to smaller amounts of OBs produced by infected first instars compared with infected third instars.

### 2.2. Laboratory Study

**Figure 2:**
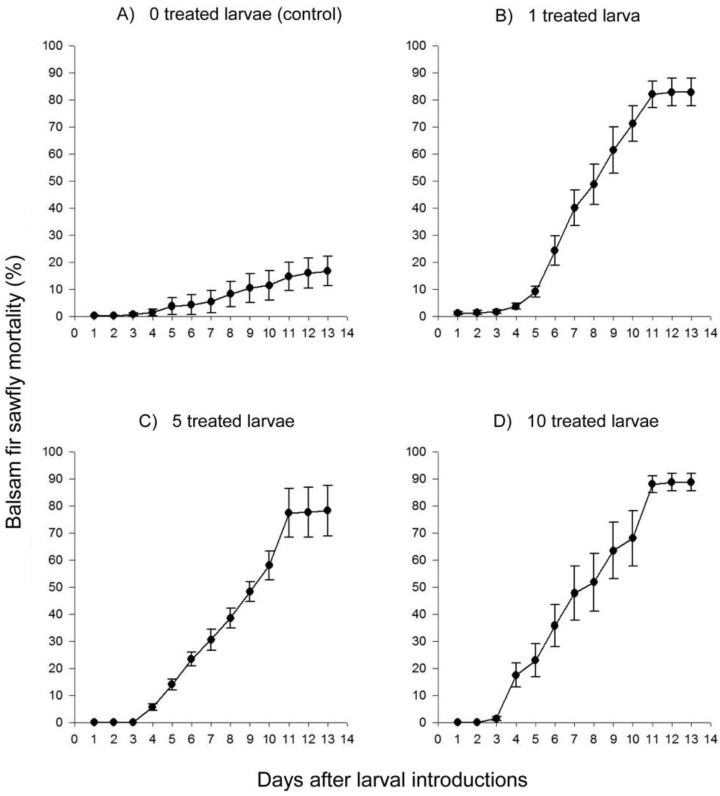
Mean (± SEM) larval mortality resulting from introducing zero (control), one, five or 10 NeabNPV-treated *N. abietis* larvae into 10 replicate groups for each treatment of first instars isolated on individual balsam fir branches in the laboratory. Each treatment group consisted of a total of 50 larvae at an equivalent density of approximately 1330 larvae/ m^2^ balsam fir foliage.

All dead larvae tested positive for NeabNPV, and all larvae that survived to the end of the experiment tested negative for NeabNPV. Both the number of treated larvae introduced into a group (Wald chi-square = 12.3, *p* = 0.006) and time since treated larvae were introduced into a group (Wald chi-square =3657.5, *p* < 0.001) influenced mortality. A low level of endemic NeabNPV resulted in levels of approximately 20% NeabNPV-induced mortality in control groups by the end of 14 d (Figure 2A). This contrasts with levels of approximately 80% NeabNPV-induced mortality, at the same time, in groups where one, five or 10 NeabNPV-treated larvae had been introduced (Figure 2B,C,D, respectively). A higher rate of mortality on the branches with infected larvae, compared with control branches, resulted from a significant interaction between time and treatment (Wald chi-square = 122.8, *p* < 0.001). 

The laboratory experiment was conducted at densities similar to those found during the peak stage of a balsam fir sawfly outbreak [10,19]. At these high densities, the number of NeabNPV-treated larvae introduced into a cohort did not significantly influence the total number of infected larvae over the course of the experiment, although it may have slightly influenced the rate at which disease spread within the cohort (Figure 2). The introduction of higher numbers of NeabNPV-treated larvae into comparatively small experimental arenas could contribute to an increased inoculum of secondarily produced NeabNPV to infect other members of the cohort. Under the high cohort densities of this experiment, even one infected larva apparently produced sufficient amounts of NeabNPV OBs in the local environment to result in the infection of many, if not all, larvae within the cohort. 

Epizootics of NeabNPV occur at peak balsam fir sawfly population outbreak densities [10,13], but it is not clear how NeabNPV epizootics are initiated and transmitted throughout balsam fir sawfly populations. Other studies on NPV transmission in sawflies have shown that epizootics are initiated from persistent environmental contamination with OBs [22,23,24,25,26,27] and subsequent spread from groups of sawfly larvae within a tree and from tree to tree [28,29,30]. The current study demonstrates how a NeabNPV epizootic might begin in balsam fir sawfly populations with the initial infection of low numbers of balsam fir sawfly larvae in isolated groups. For example, even though efforts were made to remove environmental sources of NeabNPV in the laboratory experiment, NeabNPV-induced mortality was still ~ 20% in the controls. It has been shown recently that NeabNPV infections in larvae can carry through to adult female balsam fir sawflies in midgut epithelial cells [15]. Gammabaculoviruses are not thought to be transmitted transovarially because of their restriction to sawfly midgut epithelial cells [13,14]. It is possible, however, that adult females that emerged from puparia with NeabNPV infections in their midguts may have been the source of NeabNPV contamination here. In this scenario, contamination of foliage would have occurred when infected adult females excreted NeabNPV-laden frass at the time of oviposition.

There have been relatively few studies on NPV transmission in sawflies [24,25,26,27,28,29,30,31] compared with NPV transmission in lepidopteran defoliators [17,18,32,33,34,35,36,37,38,39]. Many of the lepidopteran NPV studies have been based on investigations of the simplified model for disease transmission among invertebrates developed by Anderson and May [40,41,42]. In the lepidopteran NPV studies, it has often been noted that horizontal transmission of NPVs is not linearly density dependent, as the density dependence assumption of the Anderson and May model suggests [17,18,32,33,35,37,39]. Our results do not permit a rigorous test of this model, as we did not manipulate larval density and only tested viral transmission within and not between cohorts. Nevertheless, our results do suggest that NeabNPV transmission within larval cohorts in natural populations of balsam fir sawfly increases in proportion to the number of infected larvae. Transmission between cohorts may be highest in mid and late instars, when larvae exhibit much higher rates of movement within host tree crowns than early instars [20]. Higher balsam fir sawfly densities observed at the peak of outbreaks will lead to crowding and a greater likelihood of horizontal transmission of NeabNPV [19].

## 3. Materials and Methods

### 3.1. Balsam Fir Sawfly Life History

In Newfoundland, balsam fir sawfly eggs are laid in September and October in the current-year foliage of balsam fir trees, where they overwinter [8]. Eggs hatch and larvae emerge from late June to mid-July. Balsam fir sawfly larvae are gregarious [20] and feed on balsam fir foliage that is 1 year old and older [43,44]. Male larvae develop through five instars, over approximately 30 d, and females undergo five or six instars, completing development in about 35 d [8]. Subsequently, larvae spin cocoons on current-year foliage, pupate, and emerge as adults in late August and early September.

### 3.2. NeabNPV

*Neodiprion abietis Gammabaculovirus* (NeabNPV) was first isolated from balsam fir sawfly larvae collected from outbreak populations in western Newfoundland in 1997 [10]. Methods to mass produce NeabNPV in field populations of balsam fir sawfly larvae were developed so that field trials could be carried out to establish the efficacy of NeabNPV against populations of balsam fir sawfly larvae [10,12]. This Newfoundland isolate of NeabNPV has been registered as the biological insecticide, Abietiv™, for operational use in control programs directed against balsam fir sawflies in Canada [11,45]. The genome of this NeabNPV isolate has been sequenced and analyzed [8,46].

### 3.3. Field Study

In the first week of July 2003, first-instar balsam fir sawfly larvae were collected from a field population in an immature (tree height 5–20 m) balsam fir stand (49°12'22''N 57°33'54''W) beyond the leading edge of the balsam fir sawfly outbreak, where NeabNPV prevalence was assumed to be low. Larvae were transported to a young, precommercially thinned balsam fir stand (49°11'N 57°32'W) located approximately 7 km northwest of Deer Lake, NL where there was no history of balsam fir sawfly populations within the last 50 years [5]. Groups of 50 first-instar larvae (initial density approximately 220 larvae/ m^2^ foliage) were placed on each of five balsam fir branches (approximately 50 cm long × 45 cm wide) on each of 12 trees (60 branches, 3000 larvae total). Each branch with its larvae was covered with a muslin sleeve cage [43]. At the first or third stadium, larvae were removed from their respective groups and allowed to feed for 30 min on foliage that had been sprayed with an aqueous suspension of NeabNPV (1 × 10^2^ OBs/mL), using a 1-L aerosol spray bottle, before they were returned to the sleeve cages. Densities of zero (*i.e*., control), one or 10 of these NeabNPV-treated larvae were randomly assigned to 10 replicate populations each (*i.e.*, first or third instars, zero, one or 10 larvae returned = 60 branches), in such a way as to bring each replicate population back to a total of 50 individuals. Larvae were then allowed to develop until mid-August, when death or pupation had occurred for all members of each group. Experimental branches, still enclosed in sleeve cages, were cut outside the base of the sleeve cage with pruning shears and transported to the Canadian Forest Service Field Station at Pasadena, NL (49°01′29.9″N: 57°35′24.1″W), where they were examined for balsam fir sawfly pupae, and survival to pupation was recorded. All pupae and any remaining dead larvae on the branches or in the sleeve cages were placed in individual 1.5-mL polypropylene microcentrifuge tubes (Fisher Scientific, Fair Lawn, NJ) and stored at –20 °C for molecular probing (see below). 

### 3.4. Laboratory Study

In August 2002, balsam fir sawfly cocoons were collected from field populations in a precommercially thinned balsam fir stand (49°10’N 57°26’W) near Corner Brook, NL. This stand was outside the leading edge of the 2002 balsam fir sawfly outbreak, so NeabNPV prevalence was expected to be low. Cocoons were individually placed in 7-mL gel capsules, which were then placed in clear plastic Ziploc™ bags, then into 20-kg brown paper bags and transported by truck to laboratory facilities at the University of New Brunswick (UNB), in Fredericton, NB. Gel capsules containing cocoons were placed in a climate-controlled chamber (Conviron, Winnipeg, MB, model CMP 3032) at 18 °C, ~60% humidity, and a light regime of 18L: 6D until adult emergence. Mated adult female balsam fir sawflies can lay female (fertilized) or male (unfertilized) eggs, but unmated females lay only male eggs (arrhenotoky). To reduce the possible introduction of unwanted NeabNPV and uncertainty regarding female mating status, only unmated adult balsam fir sawfly females were individually transferred to sleeve cages on branches of young, 1–10 m tall, balsam fir trees in the UNB woodlot (45°52'N 66°32'W). To the best of our knowledge, this woodlot has never experienced an outbreak of balsam fir sawflies. After 4 d, 28 females had laid between nine to 70 eggs/female. The caged branches were left over the winter, and in the last week of May 2003, egg-bearing branches were cut with pruning shears and transported to the laboratory. Each branch was placed in a 750-mL standard mason jar filled with 500 mL of de-ionized water. Approximately 0.5 µL of commercial bleach (5.25% NaOCl) was added to each jar to discourage the growth of microorganisms. Branches were pruned in a way not to remove any eggs to 25 cm × 15 cm foliar area. Jars containing branches were placed in individual styrofoam packing trays with a 2.5-cm strip of Tree Tanglefoot Insect Barrier (Contech Enterprises Inc. Victoria, BC) around the perimeter of each tray, and trays were further separated by 5 cm to ensure that larvae could not crawl between jars. Branches were monitored and misted with distilled water daily for 13 d while waiting for the eggs to hatch. 

On 9 June 2003, when 90% of the eggs had hatched, larval densities were reduced to 50 larvae per jar of branches (initial density ~1330 larvae/m^2^ foliage) by removing or adding larvae as required. Needles with unhatched eggs were also removed at this time. Ten jars each were randomly assigned to the following treatment groups: (A) no larvae treated with NeabNPV (control); (B) one larva treated with NeabNPV; (C) five larvae treated with NeabNPV; and (D) 10 larvae treated with NeabNPV. The latter three treatments were obtained by removing one, five, or 10 larvae from the non-control groups and allowing them to feed for 30 min on foliage that had been sprayed with an aqueous suspension of NeabNPV as above. Treated larvae were then returned to their respective groups. Similarly, control groups were fed on foliage that had been sprayed with distilled water. Each day for the next 14 d, the number of living larvae was recorded for each group of branches, and clean (5-min soak in 0.25% aqueous NaOCl followed by three 15-min rinses in tap water), fresh foliage was added as required. All dead larvae were removed daily, individually placed in 1.5-mL polypropylene microcentrifuge tubes and stored at –20 °C for molecular probing. After 14 d, any surviving larvae were similarly placed in microcentrifuge tubes and frozen for probing. 

### 3.5. Molecular Probing for the Presence of NeabNPV

All retained larvae (live and dead at the time of collection) were probed for the presence of NeabNPV DNA using Renaissance™ molecular labeling and detection kits (Perkin-Elmer Life Sciences, Waltham, MA) [10]. Briefly, fluoroscein-N6-dATP-labeled DNA probes (typically between 300–600 bases) were produced using seven NeabNPV DNA/*Eco*R1fragments (3.5–5.5 kb) as templates. Individual insects were thawed and homogenized in ~ 1 µL of double-distilled water in the 1.5-µL microcentrifuge tubes in which they had been stored. A 3-µL aliquot of each sample was blotted onto Biodyne A nylon membrane (Pall Corporation, Ann Arbor, MI). Positive controls of purified NeabNPV DNA or NeabNPV OBs were also spotted onto each membrane. Membranes were soaked in denaturing solution (0.5 N NaOH, 1.5 M NaCl) and incubated at 65 °C for 30 min. Membranes were neutralized in 1.5 M NaCl, 0.5 M Tris. pH 7.0 for 1 min, soaked a further 5 min in 10 × SSC and air dried on filter paper. Target DNA was bound to the membranes by exposure to 125 mJ of UV radiation (GS Gene Linker™, Bio-Rad, Hercules, CA). Membranes were then soaked in hybridizing solution containing the labeled probe for 18 h at 65 °C. Excess probe and probe bound to non-specific DNA were removed with high stringency washes and results were recorded on BioMax ML film (Kodak, Rochester, NY). The lower detection limit for the probing protocol was 5 × 10^3^ OBs [9], implying a positive detection only for specimens where NeabNPV had replicated.

### 3.6. Statistical Analysis

For the field experiment, the effects of the initial number of NeabNPV-treated larvae (zero (control), one or 10) and the age at exposure to NeabNPV (first or third instar) on mortality were analyzed using a generalized linear model with logit links and a binomial distribution. The influence of the number of NeabNPV-treated larvae and time since treatment on mortality in the laboratory assays was also analyzed using a generalized linear model with logit link and binomial distribution. Both analyses were carried out using PASW Statistics 18, v18.0.0 (SPSS Inc. 2009, Chicago, IL).

## 4. Conclusions

The commercial product Abietiv™, which is registered in Canada for use against balsam fir sawflies, has NeabNPV as its active ingredient [11,12,45]. Abietiv is applied aerially at rates of 1–3 × 10^9^ NeabNPV OBs/ha [10,12] which are considerably lower than application rates for alphabaculovirus-based products [2]. The gregarious nature of balsam fir sawfly larval feeding, especially during early instars [20], restriction of NeabNPV infection to the larval midgut, and rapid replication and dissemination of NeabNPV OBs from infected larval guts [15,46] all contribute to the efficacy of the low application rates for Abietiv. As we have shown here, it requires only a few larvae to be infected with NeabNPV for that infection to be horizontally transmitted to other larvae within the cohort.
